# Immunophenotyping of T Cells in Lung Malignancies and Cryptogenic Organizing Pneumonia

**DOI:** 10.3390/jcm14020316

**Published:** 2025-01-07

**Authors:** Toyoshi Yanagihara, Kentaro Hata, Keisuke Matsubara, Kazufumi Kunimura, Kunihiro Suzuki, Kazuya Tsubouchi, Satoshi Ikegame, Yoshinori Fukui, Isamu Okamoto

**Affiliations:** 1 Department of Respiratory Medicine, Graduate School of Medical Sciences, Kyushu University, Fukuoka 812-8582, Japan; 2 Department of Respiratory Medicine, Fukuoka University Hospital, Fukuoka 814-0180, Japan; 3 Division of Immunogenetics, Department of Immunobiology and Neuroscience, Medical Institute of Bioregulation, Kyushu University, Fukuoka 812-8582, Japan

**Keywords:** mass cytometry, cytometry by time-of-flight (CyTOF), cancerous lymphangitis, lymphoma, cryptogenic organizing pneumonia

## Abstract

**Background:** Lung malignancies, including cancerous lymphangitis and lymphomas, can mimic interstitial lung diseases like cryptogenic organizing pneumonia (COP) on imaging, leading to diagnostic delays. We aimed to identify potential biomarkers to distinguish between these conditions. **Methods:** We analyzed bronchoalveolar lavage fluid from 8 patients (4 COP, mean age 59.8 ± 13.5 years; 4 lung malignancies including 2 cancerous lymphangitis, 1 MALT lymphoma, and 1 diffuse large B cell lymphoma, mean age 67.8 ± 4.5 years) using mass cytometry with 35 T cell markers. Data were analyzed using principal component analysis (PCA) and unsupervised Citrus clustering. **Results:** PCA of T cell marker intensities effectively separated the two groups, with IL-2Rα, PD-L2, CD45RA, CD44, and OX40 being the top discriminating markers. Citrus analysis showed a significant increase in the CD16+ CD4+ and CD16+ CD8+ T cell populations in the COP group compared to lung malignancies. **Conclusions:** Our findings reveal distinct T cell immunophenotypes in COP versus lung malignancies, particularly increased CD16+ T cells in COP, which could serve as potential diagnostic biomarkers.

## 1. Introduction

Cancerous lymphangitis is a condition characterized by the infiltration and inflammation of lymphatic vessels, caused by cancer cells disseminating from a primary location. The clinical presentation of cancerous lymphangitis is commonly pulmonary and is an indicator of advanced-stage malignancy with a poor prognosis. It accounts for approximately 6–8% of pulmonary metastases and is frequently associated with primary malignancies such as lung, breast, stomach, prostate, colon, pancreas, uterus, thyroid, cervix, and larynx carcinomas [1]. Diagnosis of cancerous lymphangitis is based on a patient’s clinical history, symptoms, and imaging findings. On chest CT imaging, smooth thickening of interlobular septa and peribronchovascular interstitium may be seen in the early stages, while nodular thickening may be observed in advanced stages. CT imaging of cancerous lymphangitis can resemble interstitial lung diseases (ILDs), such as cryptogenic organizing pneumonia (COP), leading to delayed diagnosis [2,3,4].

Lymphomas can affect the lungs as primary pulmonary lymphoma without extrapulmonary disease or as a secondary pulmonary manifestation of the known extrapulmonary or nodal disease [5]. Radiological findings in most cases include single masses, multiple nodules with peribronchovascular distribution, slow-developing consolidation, or ground-glass opacities [5]. Similar to cancerous lymphangitis, these radiological findings of pulmonary lymphomas can resemble ILDs such as COP, potentially leading to delayed diagnosis [6,7].

Organizing pneumonia is a type of lung-tissue repair mechanism that occurs following lung injury. This repair pattern can be cryptogenic or can occur in response to a specific type of lung injury. Organizing pneumonia is observed histopathologically in various clinical contexts and is classified as a type of idiopathic interstitial pneumonia since the cause is unknown. COP can be successfully treated if appropriately managed [8]. However, it is often misdiagnosed, and distinguishing COP from lung malignancies like cancerous lymphangitis and lymphomas can be challenging [1,6,7]. Useful biomarkers are, therefore, required to make an accurate diagnosis.

Mass cytometry or cytometry by time-of-flight (CyTOF) is a novel technology that utilizes inductively coupled plasma mass spectrometry to detect metal ions labeled to antibodies that specifically bind to cellular proteins. This technology allows for a comprehensive and multi-dimensional assessment of cellular composition and function [9]. In contrast to conventional flow cytometry, which has limitations such as spectral overlap and the number of parameters that can be simultaneously analyzed, mass cytometry provides a distinct advantage. It has been applied to the investigation of diverse biological systems, including immune-related diseases and cancer [10,11,12,13]. Through the analysis of large quantities of individual cells with high throughput, mass cytometry has the potential to provide a more thorough comprehension of cellular heterogeneity and signaling pathways implicated in diseases, which can aid in the detection of promising biomarkers and therapeutic targets.

The objective of this study is to employ mass cytometry to investigate bronchoalveolar lavage fluid (BALF) specimens, mainly focusing on T cells in order to differentiate pulmonary engagement in patients with pulmonary malignancies resembling ILDs and COP. Our goal is to advance our comprehension of the pathogenesis of these complications by characterizing the cellular and molecular alterations in BALF from affected individuals.

## 2. Materials and Methods

### 2.1. Patients

Patients who underwent BALF collection at Kyushu University Hospital between January 2017 and April 2022 and were newly diagnosed with cancerous lymphangitis, lymphoma, and COP were eligible for enrollment in this study. The Ethics Committee of Kyushu University Hospital approved the study (reference number 22117-00). The diagnostic criteria for cancerous lymphangitis, lymphoma, and COP were consistent with those described previously [1,5,14].

### 2.2. Mass Cytometry

Metal-tagged antibodies were either purchased from Standard Biotools (San Francisco, CA, USA) or acquired in a purified form, then labeled with metals using the MaxPar Antibody Labeling Kit (Standard Biotools), according to the manufacturer’s instructions (Table A1). Cryopreserved BALF cells were thawed in PBS, followed by staining with Cell-ID Cisplatin-198Pt (Standard Biotools #201198, diluted 1:2000 in PBS) and FcR blocking reagent (Miltenyi, Bergisch Gladbach, Germany, #130-059-901). Each CD45 antibody labeled with a different metal (as listed in Table A1) was used to barcode the cells. After washing, CD45-labeled cells were mixed and stained with the antibody cocktail (Table A2), and the amount of antibody was optimized using preliminary experiments with metal minus one. The commercially available metal-conjugated antibodies have already been validated in various studies [15,16,17,18].

For this study, we processed the samples as follows: Batch #1: All COP cases were included in this batch. Batch #2: All cases of lung malignancies were included in this batch. Following fixation with 1.6% formaldehyde and washing, the cells were suspended overnight at 4 °C in Fix and Perm Buffer containing Cell-ID Intercalator 103Rh (Standard Biotools #201103A). Cell acquisition was performed using a Helios mass cytometer at 200–300 events per second, with cells suspended in MaxPar Cell Acquisition Solution (Standard Biotools #201240) supplemented with EQ Four Element Calibration Beads (Standard Biotools #201078) at a 5:1 ratio. Using Helios software version 6.7 (Standard Biotools), the data were processed by converting to FCS format, randomizing, and normalizing based on EQ bead intensity. Subsequently, using FlowJo v10.8 software (BD Biosciences, Franklin Lakes, NJ, USA), we merged FCS files from each group and performed manual gating. The consolidated data were then analyzed using Uniform Manifold Approximation and Projection (UMAP) analysis and Citrus analysis [19] in CytoBank Premium (CytoBank Inc., Mountain View, CA, USA).

### 2.3. Data Analysis

Live cells were identified by excluding cisplatin-positive cells and doublets, followed by selecting CD45+ cells for further analysis. T cells were identified by gating for CD2+ and CD3+ cells and were subjected to UMAP, principal component analysis (PCA), and Citrus algorithms. For UMAP analysis, channels for various T cell markers, such as CD4, CD8a, CD27, CD28, CD45RA, CD45RO, and Fas, were clustered, and the parameters of numbers of neighbors and minimum distance were set to 15 and 0.01, respectively. To compare the T cell samples at a broad level, PCA was used with median expression as a univariate summary for each marker, utilizing R packages factoextra, ggplot2, and ggplotly in RStudio (version 2022.12.0 + 353 with R version 4.2.2). For the Citrus algorithm of T cells, channels for various markers were clustered, such as CD4, CD5, CD7, CD8a, CD11a, CD16, CD27, CD28, CD44, CD45RA, CD45RO, CD49d, CD57, CD69, CD226, Fas (CD95), IL-2Rα (CD25), PD-L1 (CD274), PD-L2 (CD273), PD-1 (CD279), OX40 (CD134), TIGIT, TIM3 (CD366), CTLA-4 (CD152), LAG-3 (CD223), BTLA (CD272), ICOS (CD278), ST2, CCR7 (CD197), CXCR3 (CD183), and HLA-DR. For the analysis, we set the following parameters: nearest shrunken centroid for association modeling, abundance for cluster characterization, 5% as the minimum cluster size, single-fold cross-validation, and a false discovery rate of 1%.

### 2.4. Statistical Analysis

Statistical analyses were performed using GraphPad Prism 9 software. The Citrus algorithm analysis utilized a PAMR association model with a conservative 1% FDR threshold, according to the parameters described earlier. Group comparisons were conducted using the Mann–Whitney test, while the analysis of manually gated cell proportions employed two-way ANOVA with Tukey’s post hoc multiple comparison tests. Results were considered statistically significant at *p* < 0.05.

## 3. Results

### 3.1. Patient Characteristics and Clinical Parameters

**Figure 1:**
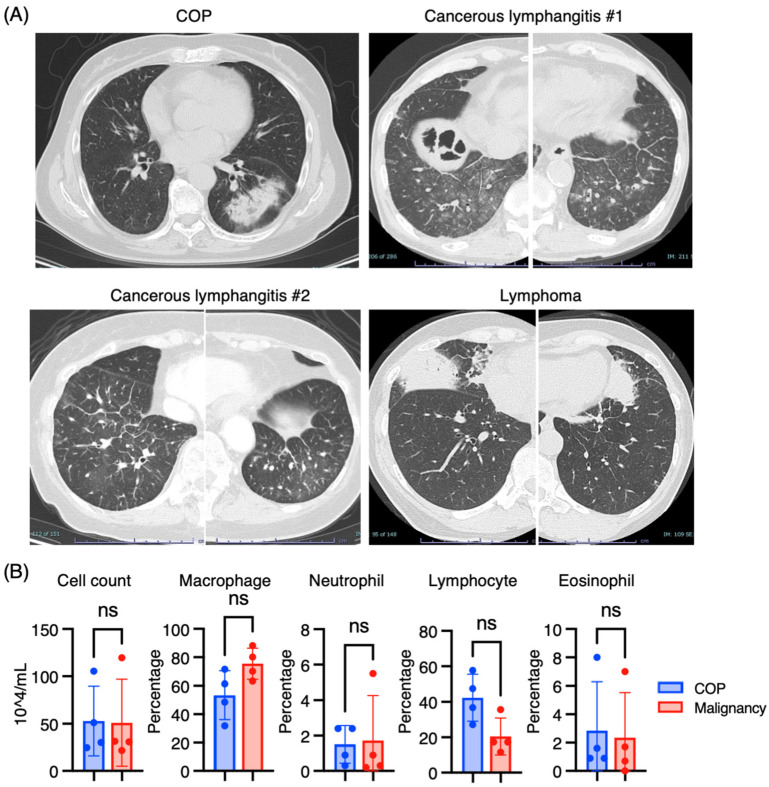
Chest CT images and BALF cell differentiation of the patients with COP and lung malignancies. (**A**) Representative CT images from patients with COP, cancerous lymphangitis, and lymphoma. # means the case number. (**B**) BALF cell counts and cell differentiation from patients with COP (*n* = 4) and lung malignancies (*n* = 4). BALF cells on cytospin slides were stained with May-Giemsa, followed by differential cell count based on their morphology under a light microscope. COP; cryptogenic organizing pneumonia, ns; non significant.

We analyzed four cases of COP and four cases of malignancies (two cancerous lymphangitides, one MALT lymphoma, and one diffuse large B cell lymphoma). The mean age of patients with COP and malignancies was 59.8 ± 13.5 and 67.8 ± 4.5, respectively (mean ± SD). Figure 1A shows representative CT images of COP with consolidation possessing a reversed halo sign, cancerous lymphangitis with thickening of interlobular septa and peribronchovascular interstitium and ground glass opacities, and MALT lymphoma with multiple consolidations in bilateral lungs. BALF cells stained with May-Giemsa showed a nonsignificant trend toward higher lymphocyte proportions in COP versus malignancies (42.3 ± 13.2% vs. 20.5 ± 10.4%) based on morphological analysis under light microscopy. (Figure 1B).

### 3.2. T Cell Differentiation in BALF from Patients with COP and Malignancies

**Figure 2:**
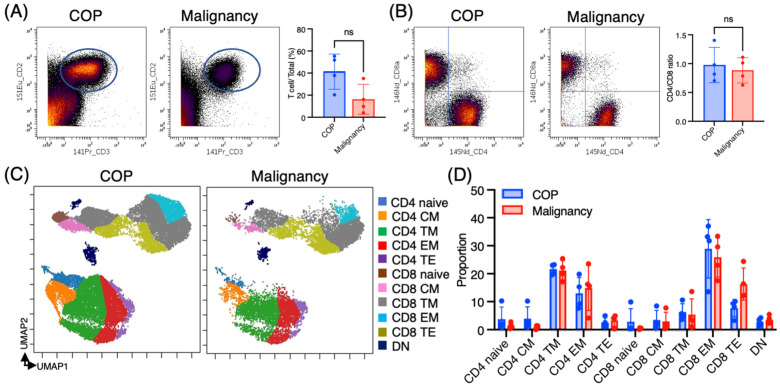
Analysis of T cell differentiation in BALF from COP and lung malignancy patients. (**A**) The dot plots depict T cells gated by CD2+ CD3+ in CD45+ bronchoalveolar lavage fluid (BALF) cells from patients diagnosed with COP (*n* = 4) and lung malignancies (*n* = 4). (**B**) The dot plots illustrate the expression of CD4 and CD8 in T cells, along with the CD4/CD8 ratio. (**C**) UMAP (Uniform Manifold Approximation and Projection) visualization of T cell differentiation states in concatenated BALF samples from both patient groups. T cell subsets are defined by the following marker combinations: naïve (CCR7+ CD45RA+), central memory (CM; CCR7+ CD45RO+ CD28+ Fas+), transitional memory (TM; CCR7− CD45RO+ CD28+ Fas+), effector memory (EM; CCR7− CD45RO+ CD28− Fas+), terminal effector (TE; CCR7− CD45RO+/− Fas−), and double negative (DN; CD4−CD8−). (**D**) The percentage of T cell subpopulations is defined in Figure 2C. Graphical plots represent individual samples. Two-way ANOVA with Tukey’s post hoc test revealed no significant differences between groups. COP; cryptogenic organizing pneumonia, ns; non significant.

First, we investigated basic information on T cells (proportion, CD4/CD8 ratio, and differential stages) in COP and malignancies. T cells were defined as CD45+ CD2+ CD3+ cells (Figure 2A). The proportion of T cells in total CD45+ BALF cells was 41.3 ± 15.9% in COP and 16.3 ± 13.5% in malignancies (Figure 2A). The CD4/CD8 ratio was 0.98 ± 0.31 in COP and 0.84 ± 0.22 in malignancies (Figure 2B). UMAP plots were generated, as shown in Figure 2C, to visually represent the differentiation of T cells in the affected lungs. Most of the T cells present in the BALF exhibited characteristics of either memory or effector T cells, while only a small subset of T cells was found to be naïve (Figure 2C,D). Although not statistically significant, there was a higher tendency for the proportion of terminal effector CD8+ T cells in malignancies compared to COP (16.3 ± 5.8% vs. 7.7 ± 3.1%).

### 3.3. Differential Expression of the T Cell Marker Revealed by PCA

**Figure 3:**
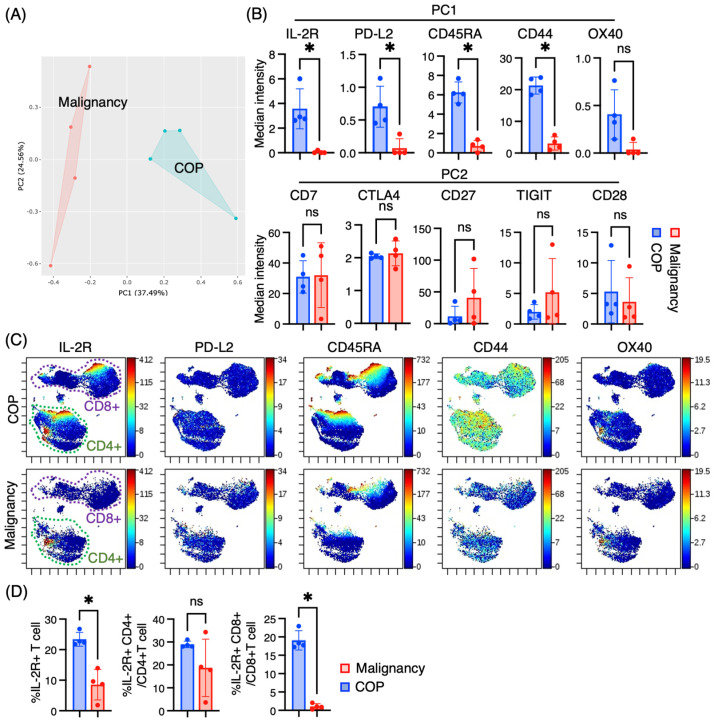
Principal component analysis (PCA) of T cell markers. (**A**) PCA of T cell markers in T cells from bronchoalveolar lavage fluid (BALF) in patients with COP (*n* = 4) and lung malignancies (*n* = 4). (**B**) The median expression of the top five markers for principal component (PC) 1 and 2. Graphical plots represent individual samples. (**C**) The UMAP plots show the expression and distribution of IL-2Rα, PD-L2, CD45RA, CD44, and OX40, the five top markers for PC1, in T cells. (**D**) The proportion of IL-2Rα+ T cells among all T cells, IL-2Rα + CD4+ T cells among CD4+ T cells, and IL-2Rα + CD8+ T cells among CD8+ T cells is shown. * *p* < 0.05. ns; non significant.

Next, we utilized PCA, a statistical method that can be used to identify patterns in complex data sets by reducing the dimensionality of the data by using the median expression for each marker on T cells in the two groups. PCA dichotomized the two groups, COP and lung malignancies (Figure 3A). The top five markers contributing to the PCA were IL-2Rα, PD-L2, CD45RA, CD44, and OX40 (Figure 3B). Regarding the top five markers of PC2, CD27, and TIGIT showed an increasing trend in malignancy, but there was no significant difference. The UMAP plots of these markers revealed that CD44 expression was distributed in the majority of T cells, IL-2Rα and CD45RA expression was restricted in some T cell populations, and PD-L2 and OX40 expression was very limited to a few T cells (Figure 3C). Concomitant with expression intensity, the proportion of IL-2Rα+ T cells, particularly in CD8+ T cells, exhibited an increase in the COP group (Figure 3D).

### 3.4. Increased CD16+ T Cell Population in BALF from Patients with COP

**Figure 4:**
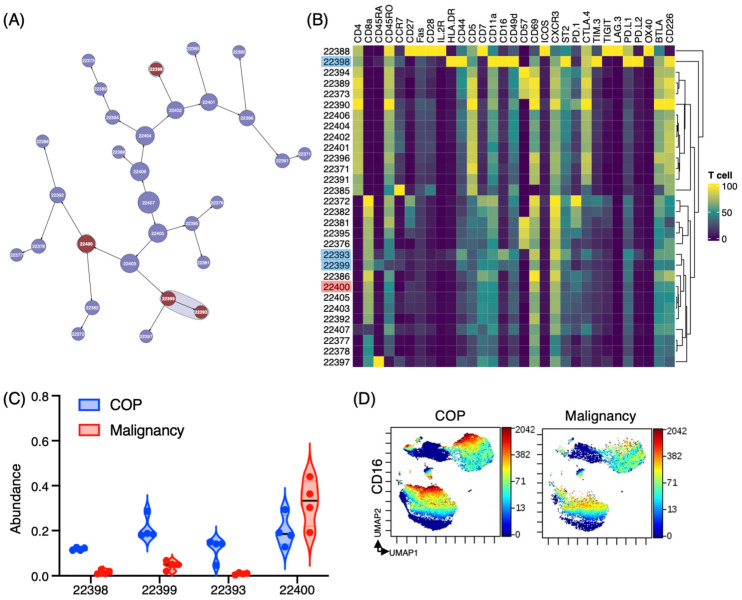
Increased CD16+ T cell population in BALF from patients with COP. (**A**) The hierarchical relationship among T cell populations in BALF from patients with COP (*n* = 4) and lung malignancies (*n* = 4) are displayed in the Citrus network tree. Clusters with significant differences are colored in red, while those without significant differences are colored in blue. The size of each circle represents the number of cells in a given cluster. (**B**) The heatmap illustrates the expression pattern of all markers across different clusters of T cells as determined by the Citrus analysis. (**C**) The Citrus-generated violin plots present the expression pattern of three distinct and differentially regulated T cell populations. Each cluster number corresponds to the number in panel (**A**). All differences in abundance are significant at a false discovery rate of less than 0.01. (**D**) The expression and distribution of CD16 in T cells are displayed on the UMAP plots.

The Citrus algorithm was employed to distinguish T cell populations with varying levels of abundance in both COP and lung malignancies. The Citrus algorithm is a widely used computational tool for identifying cell subsets and biomarkers in high-dimensional flow and mass cytometry data [19]. It offers several advantages over traditional clustering algorithms, such as the ability to detect rare cell subsets and identify biomarkers with high accuracy in an unsupervised manner [19]. Following our analysis, we detected 30 distinct clusters of T cells, out of which 4 clusters displayed significant differences between the groups (Figure 4A). Cluster #22398, which was prevalent in COP (COP vs. malignancies: 11.9 ± 0.6% vs. 1.7 ± 0.9%), was characterized by the expression of CD4+ CD16+ HLA-DR+ CD44^hi^ ST2+ TIM-3^hi^ PD-L1^hi^ PD-L2^hi^ (Figure 4B,C). Clusters #22393 (12.0 ± 4.9% vs. 1.0 ± 0.4%) and #22399 (20.9 ± 5.1% vs. 4.7 ± 2.0%), which were also prevalent in COP, consisted of CD8+ CD16+ CD44+ PD-L1+ T cells (Figure 4B,C). Cluster #22393 was also IL-2Rα positive and is considered to represent the population of IL-2Rα positive CD8 cells detected in Figure 3D. Cluster #22400, which was prevalent in lung malignancies, was CD8+ but had no specific expression pattern. UMAP plots revealed that CD16 expression was mainly observed in transient/effector memory phenotypes in CD4+ and CD8+ T cells (Figure 2C and Figure 4D).

## 4. Discussion

Our analysis revealed distinct T cell populations in BALF that differentiate between patients with COP and lung malignancies. Our analysis revealed a significant increase in CD16+ T cells in COP compared to lung malignancies in this pilot study. CD16 is an Fc receptor (FcγRIIIa) with low affinity for IgG that is typically found on natural killer cells, as well as on neutrophils and monocytes [20]. In healthy peripheral blood, only a small portion of T cells express CD16 [21]. In a recent study by Georg et al., the severity of

COVID-19 was found to be linked to CD16+ T cells that were highly activated and displayed enhanced cytotoxic capabilities [22].

T cells expressing CD16 facilitate degranulation and cytotoxicity through an immune-complex-regulated, TCR-independent approach. Additionally, CD16+ T cells derived from COVID-19 patients induced damage to microvascular endothelial cells and prompted the secretion of monocyte and neutrophil chemoattractants [22]. Georg et al. further identified that severe COVID-19 led to elevated production of C3a, which, in turn, stimulates CD16+ cytotoxic T cells. The presence of activated CD16+ T cells and plasma concentrations of complement proteins preceding C3a were linked to lethal COVID-19 outcomes, underscoring the pathological significance of heightened cytotoxicity and complement activation in COVID-19 [22]. These findings raise questions about the possible role of complement activation in COP and whether the observed increase in CD16+ T cells in COP patients might be linked to similar mechanisms. A recent study showed that lung epithelial cells could produce a C3 component, and this local complement has a pivotal role in innate immune defense [23]. We speculate that the excessive complement produced by lung epithelial cells could induce the activation of CD16+ T cells and lead to the cause of COP. The investigation of localization of the increased CD16+ T cells in COP would add insight into the pathogenesis of COP. Further investigation into the interplay between complement activation and CD16+ T cells in COP could help elucidate the pathological processes involved in this disease.

In addition to COVID-19, we recently discovered an expansion of the CD16+ T cell population in BALF from patients with *Pneumocystis jirovecii* pneumonia (PCP) compared to cytotoxic drug-induced ILD and immune-checkpoint inhibitor-associated ILD [24]. Notably, the proportion of CD16+ T cells was the highest in a fatal case of PCP. Given these findings of increased CD16+ T cells in both COVID-19 and PCP, increased CD16+ T cells in COP suggest that initiation of COP could be an infection of undetected microorganisms. It has been suggested that certain viral or bacterial infections might contribute to the development of COP by inciting a robust immune response that causes lung injury, although COP should be diagnosed only after the exclusion of identifiable infection [25].

We identified that these increased CD16+ T cells in COP were HLA-DR^+^ CD44^hi^ ST2^+^ TIM-3^hi^ PD-L1^hi^CD4 + T cells, which were the unique T cell populations that have not been reported previously. We can, however, assume some potential functions by analyzing the individual markers. HLA-DR expression on T cells can be indicative of an activated state as it is upregulated upon activation [26]. ST2 is a receptor for the cytokine IL-33 and is associated with T-helper 2 (Th2) cell responses. ST2 expression in T cells can be linked to the regulation of inflammation and tissue repair [27]. TIM-3 is an inhibitory receptor expressed in T cells and other immune cells [28]. Its expression is often associated with T cell exhaustion or dysfunction, particularly in the context of chronic infections or cancer [28]. PD-L1 is a protein involved in immune checkpoint regulation [29]. When expressed in T cells, it can interact with the PD-1 receptor on other T cells or immune cells, suppressing T cell activation and proliferation [29]. PD-L2′s role in T cell responses is disputed as it has both coinhibitory and costimulatory effects [29]. Besides interacting with PD-1, PD-L2 binds to RGMb, a co-receptor present in immune cells that regulates respiratory tolerance [29]. In an asthma mouse model, a mutant form of PD-L2 (K113S) lacking PD-1 interaction promotes Th1 polarization and suppresses Th2-mediated responses, acting as a costimulator for CD4+ T cell responses [30]. This study demonstrates the expression of PD-L2, an immune checkpoint molecule with a poorly understood role, and may shed some light on the role of PD-L2 in lung disease. Considering the functions of these individual markers, CD16+ HLA-DR+ ST2+ TIM-3+ PD-L1+ PD-L2+ CD4+ T cells may represent a unique subset of T cells exhibiting activation and cytotoxicity features with immune checkpoint modulation and potential exhaustion. Further research is needed to identify specific antigens recognized by TCR and whether this T cell population has a pathological function in developing COP using in vitro experiments.

On the other hand, PCA of individual marker intensities effectively discriminated between the two groups, COP and lung malignancies. The top five markers contributing to the PCA were IL-2Rα, PD-L2, CD45RA, CD44, and OX40, which exhibited elevated expression in COP. We acknowledge that markers like OX40, expressed on a minor proportion of T cells with a median expression below 1, might not be directly relevant to T cell function or disease pathogenesis. However, we believe that this unsupervised approach has the potential to uncover unexpected yet significant findings. Furthermore, even if we cannot directly associate these marker differences with significant functional implications, they could still serve as diagnostic markers within a big data framework, especially in cases where distinguishing between COP and malignancies remains uncertain. Regarding IL-2Rα, we analyzed IL-2Rα+ proportions among T cells and discovered that the proportion of IL-2Rα+ T cells, especially IL-2Rα + CD8+ T cells, was elevated in the COP group (Figure 3D). Previous studies have shown their significance in immune responses. For instance, IL-2Rα expression on CD8+ T cells has been associated with enhanced survival and effector function in antigen-specific responses [31]. In chronic viral infections, these cells demonstrate increased activation states and participate in pathogen-specific immune responses [32]. Though we currently lack functional data on these IL-2Rα + CD8+ T cells, it is plausible that these cells, which seem to be part of cluster #22393 expressing CD16 +, could play a role in COP pathogenesis.

This pilot study has several key limitations: it analyzed a limited number of patients, lacked healthy control samples, and was conducted retrospectively. These factors may result in missing clinical data for certain cases and potential selection bias as only patients who underwent bronchoalveolar lavage were eligible for enrollment in the study. These initial findings lay the groundwork for more comprehensive future investigations that will include expanded patient cohorts and proper control groups. Verification of increased CD16+ T cells in COP in lung tissues by immunohistochemistry would be desirable. Despite these limitations, our findings contribute to a better understanding of the T cell populations in COP and lung malignancies, paving the way for further exploration of their potential roles in the pathogenesis of these conditions.

## 5. Conclusions

In conclusion, this study highlights the distinct T cell immunophenotypes present in bronchoalveolar lavage fluid from patients with COP and lung malignancies. The significant increase in CD16+ T cell populations in COP and the successful discrimination between the two groups using principal component analysis emphasize the potential of these identified markers in aiding diagnosis and understanding the underlying immune mechanisms of these diseases.

Further research is needed to validate these findings in larger cohorts and to elucidate the specific roles and functions of these T cell populations in disease pathogenesis. The development of novel diagnostic tools and therapeutic strategies targeting these immune cells could potentially improve the accuracy and timeliness of diagnosis, ultimately benefiting patients with COP and lung malignancies.

## Data Availability

Raw data from this study will be provided by the corresponding author upon reasonable request.

## Abbreviations

BALF	bronchoalveolar lavage fluid
COP	cryptogenic organizing pneumonia
COVID-19	coronavirus disease 2019
CyTOF	cytometry by time-of-flight
FDR	false discovery rate
ILD	interstitial lung disease
MALT	Mucosa-associated lymphoid tissue
PBS	phosphate-buffered saline
PCA	principal component analysis
PCP	pneumocystis pneumonia
TCR	T cell receptor
UMAP	Uniform manifold approximation and projection

## Appendix A. Mass Cytometry Antibody Panels

**Table A1:** For CD45 barcoding.

Label	Target	Product_ID	Clone	Amount (uL)
89Y	CD45	3089003B	HI30	1
106Cd	CD45	3106001C	HI30	1
110Cd	CD45	3110001C	HI30	1
112Cd	CD45	3112001C	HI30	1
116Cd	CD45	3116001C	HI30	1
196Pt	CD45	3196001C	HI30	1

**Table A2:** For antibody cocktail.

Label	Target	Product ID	Clone	Amount (μL)
141Pr	CD3	3141019B	UCHT1	1
142Nd	CD11a	3142006B	HI111	0.5
143Nd	CD5	3143007B	UCHT2	1
145Nd	CD4	3145001B	RPA-T4	1
146Nd	CD8a	3146001B	RPA-T8	1
147Sm	CD7	3147006B	CD7-6B7	1
148Nd	PD-L1	3148017B	29E.2A3	1
149Sm	IL-2Rα	3149010B	2A3	1
150Nd	OX40	3150023B	ACT35	1
151Eu	CD2	3151003B	TS1/8	1
152Sm	Fas	3152017B	DX2	1
153Eu	TIM-3	3153008B	F38-2E2	1
154Sm	TIGIT	3154016B	MBSA43	1
155Gd	PD-1	3155009B	EH12.2H7	1
156Gd	CXCR3	3156004B	G025H7	1
158Gd	ST2	Labeling (201158A)	(R&D AF523)	1
159Tb	CCR7	3159003A	G043H7	1
160Gd	CD28	3160003B	CD28.2	1
161Dy	CTLA-4	3161004B	14D3	1
162Dy	CD69	3162001B	FN50	1
163Dy	BTLA	3163009B	MIH26	1
164Dy	CD45RO	3164007B	UCHL1	1
165Ho	LAG-3	3165037B	11C3C65	1
166Er	CD44	3166001B	BJ18	1
167Er	CD27	3167002B	O323	1
168Er	ICOS	3168024B	C398.4A	1
169Tm	CD19	3169011B	HIB19	1
170Er	CD45RA	3170010B	HI100	1
171Yb	CD226	3171013C	DX11	1
172Yb	PD-L2	3172014B	24F.10C12	1
173Yb	HLA-DR	3173005B	L243	0.5
174Yb	CD49d	3174018B	9F10	1
175Lu	CD14	3175015B	M5E2	2
176Yb	CD57	3176019B	HCD57	1
209Bi	CD16	3209002B	3G8	1
